# Image Motion Measurement and Image Restoration System Based on an Inertial Reference Laser

**DOI:** 10.3390/s21103309

**Published:** 2021-05-11

**Authors:** Ronggang Yue, Humei Wang, Ting Jin, Yuting Gao, Xiaofeng Sun, Tingfei Yan, Jie Zang, Ke Yin, Shitao Wang

**Affiliations:** 1 Institute of Remote Sensing Satellite, CAST, Beijing 100094, China; beijing2008-v@126.com (R.Y.); wywanghumei@163.com (H.W.); 13521842335@163.com (T.J.); gyting1214@163.com (Y.G.); trinight@gmail.com (X.S.); m13401155010@163.com (K.Y.); 2 Beijing Institute of Spacecraft Environment Engineering, CAST, Beijing 100094, China; yantf511@sina.com; 3 Beijing Institute of Spacecraft System Engineering, CAST, Beijing 100094, China; zj13520350752@163.com

**Keywords:** inertial reference laser, micro vibrations, image motion, image restoration, remote sensing satellite

## Abstract

Satellites have many high-, medium-, and low-frequency micro vibration sources that lead to the optical axis jitter of the optical load and subsequently degrade the remote sensing image quality. To address this problem, this paper developed an image motion detection and restoration method based on an inertial reference laser, and describe edits principle and key components. To verify the feasibility and performance of this method, this paper also built an image motion measurement and restoration system based on an inertial reference laser, which comprised a camera (including the inertial reference laser unit and a Hartmann wavefront sensor), an integrating sphere, a simulated image target, a parallel light pope, a vibration isolation platform, a vibration generator, and a 6 degrees of freedom platform. The image restoration principle was also described. The background noise in the experiment environment was measured, and an image motion measurement accuracy experiment was performed. Verification experiments of image restoration were also conducted under various working conditions. The experiment results showed that the error of image motion detection based on the inertial reference laser was less than 0.12 pixels (root mean square). By using image motion data to improve image quality, the modulation transfer function (MTF) of the restored image was increased to 1.61–1.88 times that of the original image MTF. The image motion data could be used as feedback to the fast steering mirror to compensate for the satellite jitter in real time and to directly obtain high-quality images.

## 1. Introduction

With the increasing application of space technologies, the requirements for the function and performance of spacecrafts have also become increasingly strict. In response to these requirements, a series of high-resolution optical remote sensing satellites with large apertures have been developed, such as GeoEye-1 [1], WorldView-4 [2], and GF-4 [3].

However, satellites have multiple frequency disturbance sources, such as the solar wing driving mechanism, antenna driving mechanism, control moment gyroscope, thruster, and refrigerator [4]. Coupled with the attitude instability of the satellite, these sources cause on-orbit flutter, which covers a wide band of frequency, from extremely low to high frequencies of thousands of Hz [5,6,7,8]. All of these factors will lead to the jitter of the optical axis of the optical payload and move or rotate the imaging focal plane, thereby reducing the quality of the remote sensing images [9,10,11,12]. The NASA Goddard Space Center has revealed that the energy of flutter is mainly concentrated in the middle- and low-frequency regions [13]. The flutter can be partly eliminated by setting a vibration isolation mechanism [14].

To measure the effect of flutter on imaging, a variety of image motion measurement methods have been proposed. The method based on joint transform correlator (JTC) has been widely used in camera image motion measurement [15,16,17,18,19,20,21,22,23]. The authors in [24] proposed an approach to restore a motion-blurred image in real time using optoelectronic hybrid joint transform correlation. This method requires several detection equipment, including a high-speed charge coupled device (CCD) camera and optical JTC. The two adjacent images measured by the high-speed CCD are processed by the JTC to obtain the corresponding image motion, which, in turn, is fed back to the image stabilization mirror to ensure relative stability between the imaging and CCD, as well as to achieve excellent imaging. In this method, the measuring device is placed at the focal plane, and the image motion is measured with a high accuracy and speed.

The authors in [25,26,27,28,29,30] adopted an image motion detection method based on remote sensing images. In the camera exposure process, a high-speed camera was used to record multiple frames of consecutive images, and the displacement between adjacent frames was measured using a registration algorithm to realize the image motion measurement. The point spread function (PSF) for the image restoration was constructed based on image motion, and image restoration was carried out via post-processing. Despite accurately measuring the real state of image motion at the focal plane, this method could not measure the image motion at the blind spot frequency, and demonstrated hysteresis [31].

Both the image motion measurement method based on JTC and the image motion detection method based on remote sensing images require a rich image level of the object to be photographed and a high signal-to-noise ratio [17]. Ensuring accuracy becomes difficult when the remote sensing image is smooth or the signal-to-noise ratio is low.

In addition, reference [32] proposed a system to measure image motion using a position sensitive detector (PSD), which depends on a strong point light source placed around the object to be photographed. Reference [33] measured image motion using inertial measurement sensors, but the line of sight could not be measured directly.

To address the above problems, this paper proposes a high-precision image motion measurement method based on an inertial reference laser. This method depends on neither the characteristics of the ground scenery nor the lighting conditions. The reference provided by this method ensures that the image motion measurement system can work effectively all day under any weather condition. The PSF is obtained by normalizing the image motion data, and the obtained PSF is used for restoration in order to obtain an excellent image.

The rest of this paper is organized as follows. Section 2 introduces the principle of the proposed method and its key component, namely, the inertial reference laser unit. Section 3 introduces the corresponding experimental verification system and measures the background noise in the experimental environment. Section 4 explains the image restoration algorithm used in this paper. Section 5 verifies the proposed image motion measurement method and verifies its high accuracy. The paper ends with a presentation of the image restoration results and an evaluation of the effect of image restoration based on the modulation transfer function (MTF).

## 2. Principle of Image Motion Measurement Based on Inertial Reference Laser

The image motion measurement method based on inertial reference laser relies on a laser reference system with an inertial sensor. The reference laser and imaging light are introduced into the optical system by the same path, and the image motion information can be measured directly and used to drive the image stabilization mirror for real-time compensation. Based on the available image motion information, the image is restored via post-processing to reduce the impact of chatter on the image quality.

**Figure 1:**
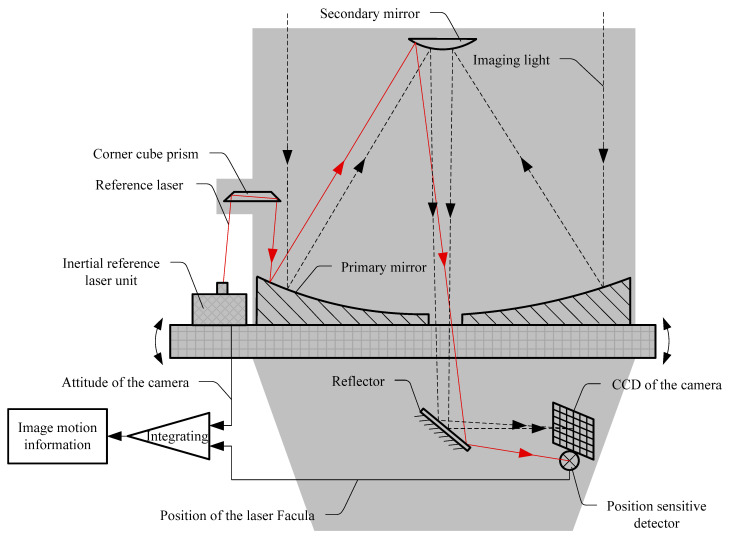
Principle of the image motion measurement method based on an inertial reference laser.

The principle of the image motion measurement method based on an inertial reference laser is illustrated in Figure 1. Specifically, a reference laser beam is emitted by the inertial reference laser unit and introduced into the camera through the corner cube prism. The reference beam and imaging light pass through the same optical path (primary mirror, secondary mirror, and image stabilizing mirror, etc.) and then reach the position sensitive detector (PSD) located at the edge of and rigidly fixed to the camera focal plane. The PSD detects the jitter information of the reference laser beam, carries the motion information of the reference beam relative to the focal plane, and then fuses the camera attitude information measured by the inertial reference laser unit. This integrated information controls the image stabilization mirror through closed-loop feedback, and the image stabilization mirror compensates for the effects of flutter on imaging through fast motion in order to obtain excellent remote sensing images. Meanwhile, based on the available image motion information, image restoration software is used to restore the degraded images and to reduce the impact of on-orbit flutter on the imaging.

**Figure 2:**
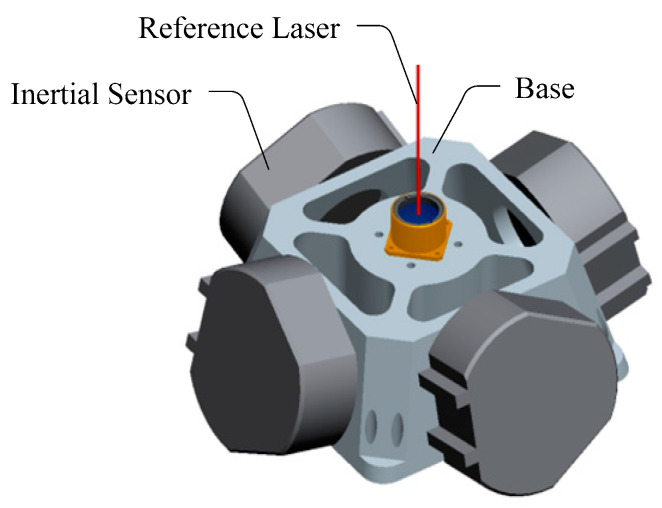
Model of the inertial reference laser unit.

As shown in Figure 2, the inertial reference laser unit mainly includes a high-precision inertial sensor and a laser source. The sensitive axes of the two-axis inertial sensors are located perpendicular to each other and are used to accurately measure the attitude change and jitter of the camera in two vertical directions within the integration time. The laser source is used to emit a reference laser beam. Given that the laser is rigidly connected to the inertial sensor, the absolute direction of the laser beam in the inertial space can be accurately measured.

## 3. Image Motion Measurement System and Experimental Environment

### 3.1. Image Motion Measurement Experimental System

**Figure 3:**
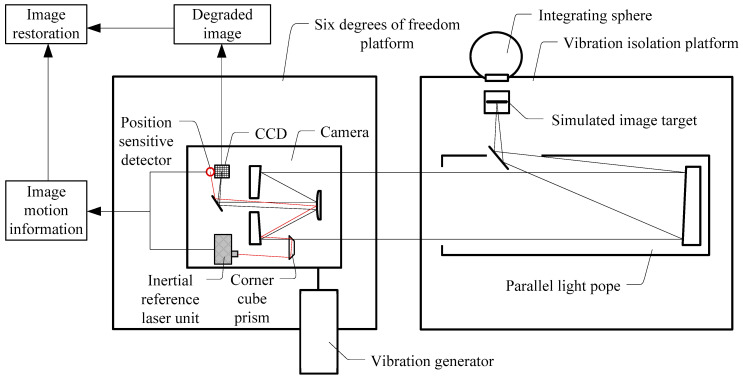
Schematic diagram of the image motion measurement system based on an inertial reference laser.

The principle of the image motion measurement experimental system based on an inertial reference laser is shown in Figure 3. The simulated image target was placed on the focal plane of the collimator to simulate a target at infinity. After being illuminated by the integrating sphere, the image target was imaged on the CCD by the camera optical system. The parallel light pope and image target were installed on a vibration isolation platform to isolate the influence of ground micro vibrations and to ensure the stability of the target light. The camera system (including the inertial reference laser unit) was installed on a 6 degrees of freedom platform to simulate the free boundary state of the camera on orbit, and the simulated micro vibrations generated by the vibration exciter were applied to the camera.

**Figure 4:**
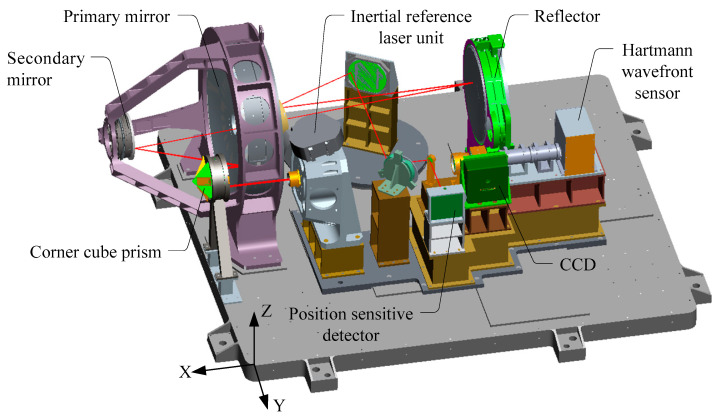
3D model of the camera.

The main lens of the camera used in the experiment had an optical aperture of 330 mm and focal length of 3300 mm. The CCD resolution was 1024 × 1024 pixels with a pixel pitch of 12 μm. Figure 4 shows that the camera was integrated with an inertial reference laser unit, a corner cube prism, and a PSD. To verify image motion measurement accuracy, an additional Hartmann wavefront sensor was integrated, which could directly measure image motion by imaging the sub-aperture image at a high speed and by calculating the offset of each frame. The root mean square (RMS) value of the image motion measurement accuracy of the Hartmann wavefront sensor was higher than 0.1 pixels.

### 3.2. Measurement of Background Noise in the Experiment Environment

The image motion measurement experiment based on an inertial reference laser depends on a precise measurement system. To reduce measurement error, the stability of the parallel light pope and image target system should be ensured. Therefore, this system was placed on a vibration isolation platform to isolate the micro vibrations from the ground. Before the experiment, the angular displacement noise around the *Y*- and *Z*-axes direction of the working face of the vibration isolation platform was measured using a high-precision angular displacement sensor (the definition of the coordinate system is shown in Figure 4). The measurement conditions were the same as those during the experiment, that is, the integrating sphere fan was kept on, the rest of the surrounding vibration experiment was stopped, the ventilation devices in the experimental area were turned off, and the shading curtains were closed.

**Figure 5:**
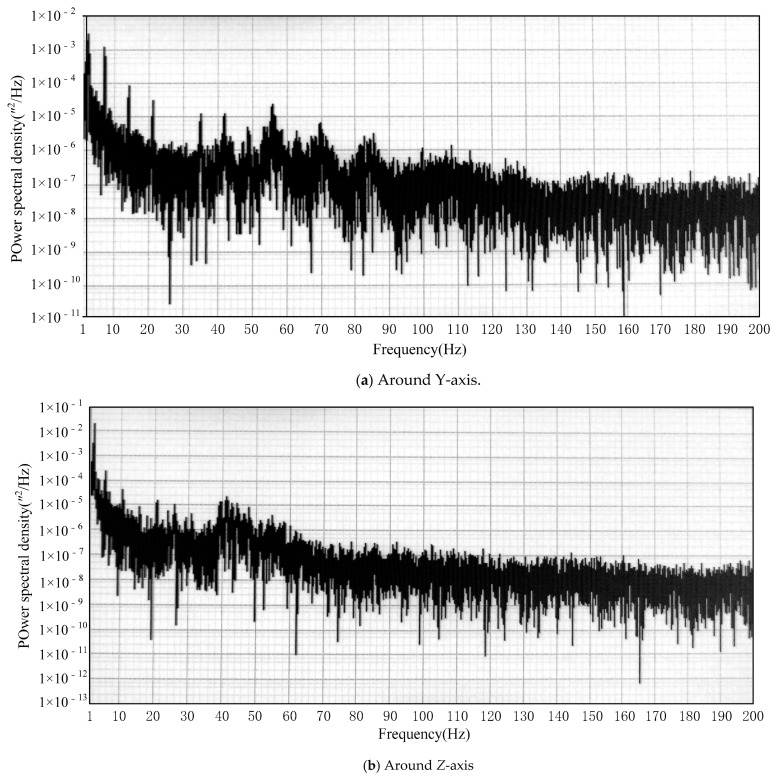
Power spectral density curve of the background noise of the vibration isolation platform.

Figure 5 shows the micro vibration power spectral density curves measured in two directions under the aforementioned conditions, and Table 1 shows the corresponding RMS noise. The background noise of the vibration isolation platform within the 1–200 Hz range did not exceed 0.037″ and was less than 1/20 pixels, while in the 3–200 Hz frequency range, the background noise did not exceed 0.014″ and was less than 1/50 pixels, thereby meeting the accuracy requirements of the experiment.

## 4. Principles of Image Restoration

Non-blind restoration algorithms have made considerable progress in the field of image restoration. Several representative non-blind restoration algorithms have achieved ideal image restoration effects in their respective application fields, such as the Wiener filtering algorithm [34], total variation regularized image restoration algorithm [35,36,37], hybrid spatio-spectral total variation image restoration algorithm [38],image restoration algorithm based on the natural image gradient distribution model [39], and fast non-blind restoration algorithm based on natural image gradient constraints [40]. The authors in [41] proposed a local piecewise regularization Richardson Lucy (RL) method and constructed a new regularization term that effectively controlled the noise and edge ringing effect in the restored image.

According to the theory of linear systems, the image degradation process can be modeled as follows:

(1) g(x,y)=f(x,y)×h(x,y)+n(x,y)

where *g*(*x, y*) is the degraded image; *f*(*x, y*) is the clear image; *h*(*x, y*) is the degeneration transfer function of the imaging system, namely, the degradation point spread function PSF; and *n*(*x, y*)represents noise.

According to the image degradation model, after the image degradation, PSF is reconstructed based on the available measurement information, the image can be restored directly using algorithms such as the deconvolution method, Wiener filtering method, or RL method. The frequency spectrum of the restored image can be formulated as:

(2) $$F\left(u\right)=\frac{G\left(u\right)}{H\left(u\right)}-\frac{N\left(u\right)}{H\left(u\right)}$$

where *G*(*u*) is the degraded image, *H*(*u*) is the degraded PSF, and *N*(*u*) is the Fourier transform of noise. The local piecewise RL algorithm was used for the image non-blind restoration, that is, based on the RL algorithm, a new regularization term was constructed to suppress noise and ringing and to improve the quality of image restoration.

The Bayesian maximum posterior probability of the restored image can be formulated as follows:

(3) $$f=argmin_{f}\{-lnP[(g|f)]-ln[P\left(f\right)]\}$$

The noise in the imaging process mainly includes readout noise and photoelectric shot noise. The readout noise is independent of the signal and follows Gaussian distribution. The photoelectric shot noise is related to the signal, which is the photoelectric noise caused by the photoelectric conversion process of the sensor and obeys Poisson distribution. Because a CCD imaging system with a low readout noise was used in the test, the noise of the image data approximately conforms to the Poisson noise model, that is, ***P*** (***g***|***f***) approximately conforms to the Poisson noise model, then:

(4) $$-ln[P(g|f)]=\sum_{i}\{{\left(hf\right)}_{i}-g_{i}ln[\left(hf{)}_{i}\right]+ln[\left(g_{i}\right)!]\}$$

where *i* is the pixel index.

To construct the regular term −ln[P(f)], a spatial neighborhood system ***S*** was initially defined, followed by a series of displacement matrix $C_{k}^{j}\;\left(j=1,2,\cdots \right)$, in order for the result of $C_{k}^{j}f$ to move the entire image for $\left|k_{j}\right|$ pixels along the direction of *K_j_*, which represents the vector from the center pixel to the other pixels in ***S***. Therefore, the regular term can be expressed as:

(5) $$J_{r}\left(f\right)=-ln[P\left(f\right)]=f^{T}Mf$$

(6) $$M=\underset{j}{\sum }\left(I-C_{k}^{j}\right)$$

where *I* is the identity matrix. Assuming that λ is the regularization coefficient, Equations (5) and (6) can be substituted into Equation (3), then:

(7) $$f=argmin_{f}\left(\underset{i}{\sum }\left\{{\left(hf\right)}_{i}-g_{i}ln\left[{\left(hf\right)}_{i}\right]\right\}+\frac{\lambda }{2}f^{T}Mf\right)$$

By using expectation–maximization (EM) algorithm, Equation (7) can be transformed into the following iterative equation:

(8) $$f^{t+1}=\frac{f^{t}}{1+{\frac{\lambda }{2}\frac{dJ_{r}\left(f\right)}{df}|}_{f^{t}}}\cdot \left(h^{T}\frac{g}{hf^{t}}\right)$$

where ***t*** is the number of iterations.

Given that $\frac{dJ_{r}\left(f\right)}{df}=\left(M^{T}+M\right)f$ and ***M*** are a symmetric matrix, $\frac{dJ_{r}\left(f\right)}{df}=2Mf$. Equation (8) can be simplified as:

(9) $$f^{t+1}=\frac{f^{t}}{1+\lambda Mf^{t}}\cdot \left(h^{T}\frac{g}{hf^{t}}\right)$$

where $Mf^{t}=\underset{j}{\sum }\left(f^{t}-C_{k}^{j}f^{t}\right)$.

The regular term $J_{r}\left(f\right)\;$ takes more pixels in neighborhood ***S*** into account, thereby allowing the RL algorithm to accurately locate discontinuous points in the image. Given that $J_{r}\left(f\right)$ imposes a heavy penalty factor on the details in the image, the following piecewise power function was used to modify this term:

(10) $$f\left(x\right)=\{\begin{array}{c} {\left(x/255\right)}^{scale}\;if\;0\leq t_{1}\leq \left(x/255\right)\leq t_{2}\\ \;0\;else \end{array}$$

where scale∈[−1,1], and *t*_1_ and *t*_2_ are two thresholds. Replace $Mf^{t}$ in Equation (9) with $f\left(Mf^{t}\right)$, which represents the point-to-point operation of vector $Mf^{t}$. The following RL algorithm for local segment regularization is then obtained:

(11) $$f^{t+1}=\frac{f^{t}}{1+\lambda f\left(Mf^{t}\right)}\cdot \left(h^{T}\frac{g}{hf^{t}}\right)$$

To further protect the edge information in the image, the residual deconvolution algorithm is used for the image restoration. The result of the regulated RL algorithm with local segmentation is denoted by ***f***_*p*_. The remaining blurred image is modeled as

(12) $$\Delta g=g-hf_{p}$$

The local piecewise regularization RL algorithm used in image restoration involves the following steps:

(1) Adopt the local piecewise regularization RL algorithm to obtain the preliminary result ***f***_*p*_; and

(2) Calculate the remaining blurred image $\Delta g=g-hf_{p}$, and then use the local piecewise regularization RL algorithm to obtain the remaining image Δf. The final restored image is $f_{p}+\Delta f$.

## 5. Accuracy Verification for Image Motion measurement

To verify the accuracy of the image motion measurement based on an inertial reference laser, the measurement data were compared with those of the Hartmann wavefront sensor. The Hartmann wavefront sensor obtained an accuracy better than 0.1 pixel (RMS) threshold for accuracy verification. The experiment process is summarized as follows.

(1) Under the condition of a constant frequency or random vibrations, a vibration exciter was used to excite the camera along the Y axis;

(2) The corresponding measurement data were recorded using the laser gyroscope, PSD, and Hartmann wavefront sensor;

(3) The measurement data from the laser gyroscope and PSD were fused to obtain the image motion measurement curve based on an inertial reference laser.

(4) The data measured by the Hartmann sensor were processed to obtain another curve.

**Figure 6:**
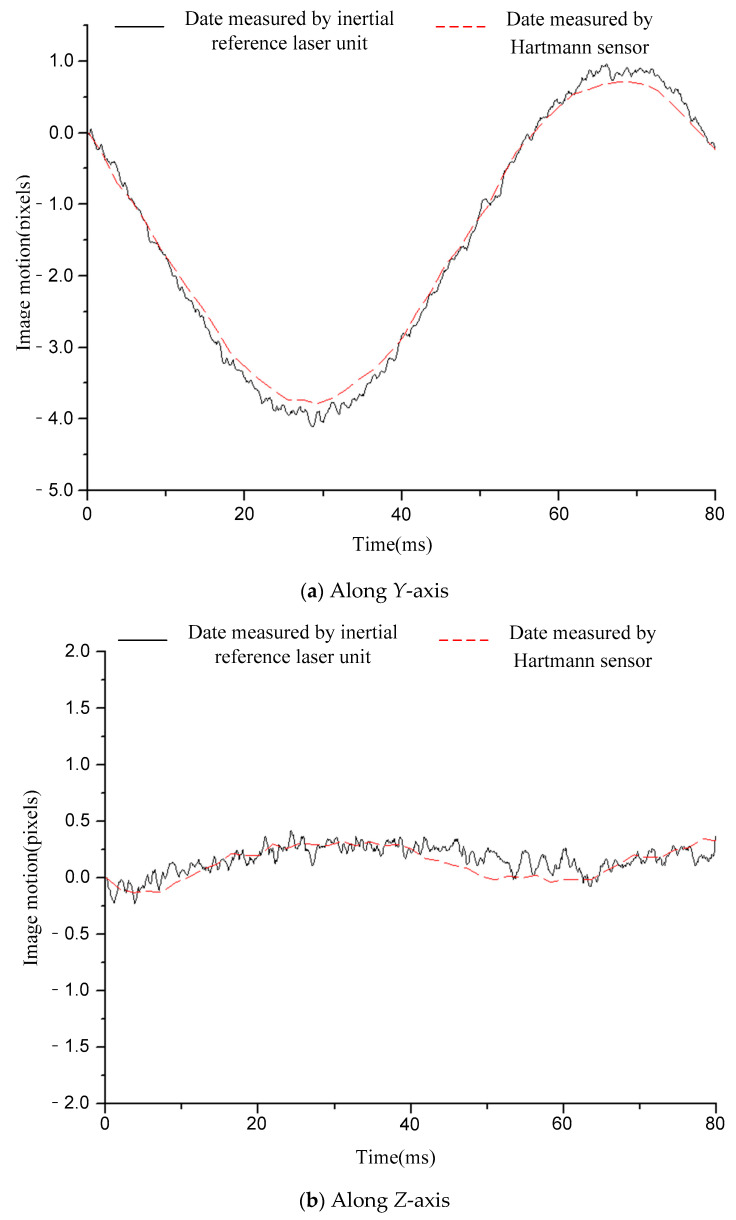
Comparison of the image motion measurement curves under the condition of 12.5 Hz constant frequency sinusoidal excitation.

**Figure 7:**
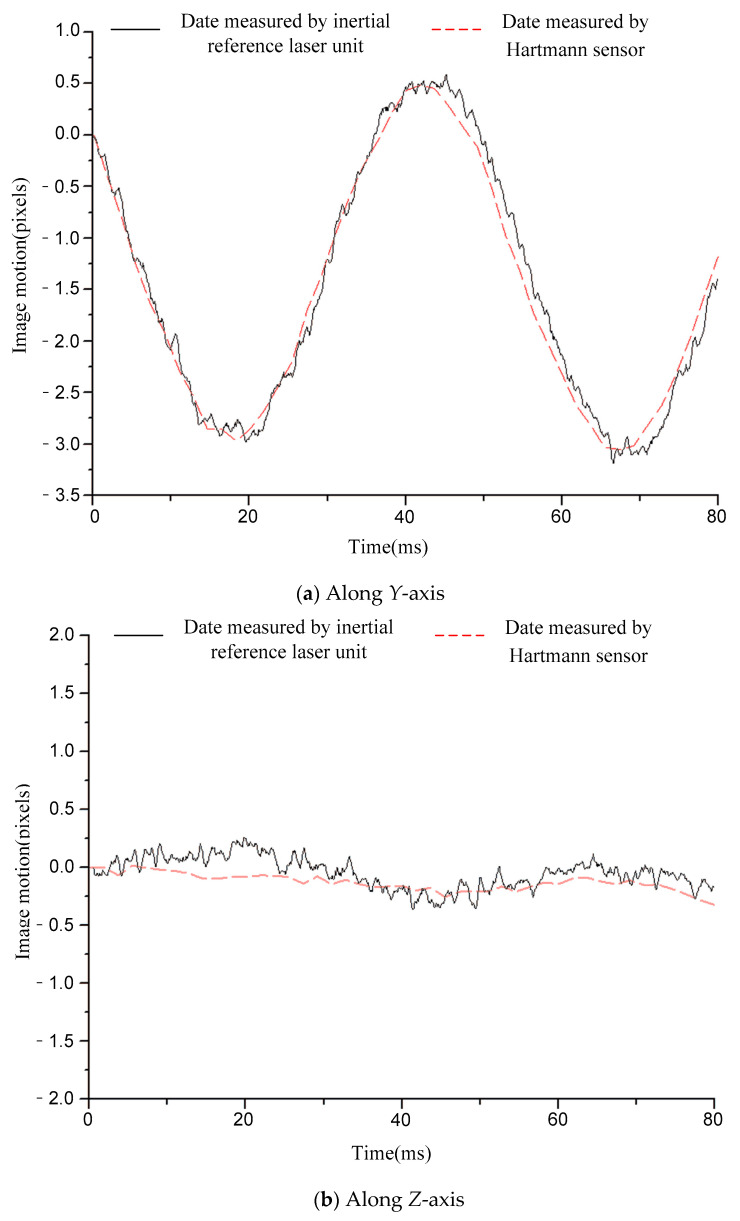
Comparison of image motion measurement curves under the condition of 20 Hz constant frequency sinusoidal excitation.

**Figure 8:**
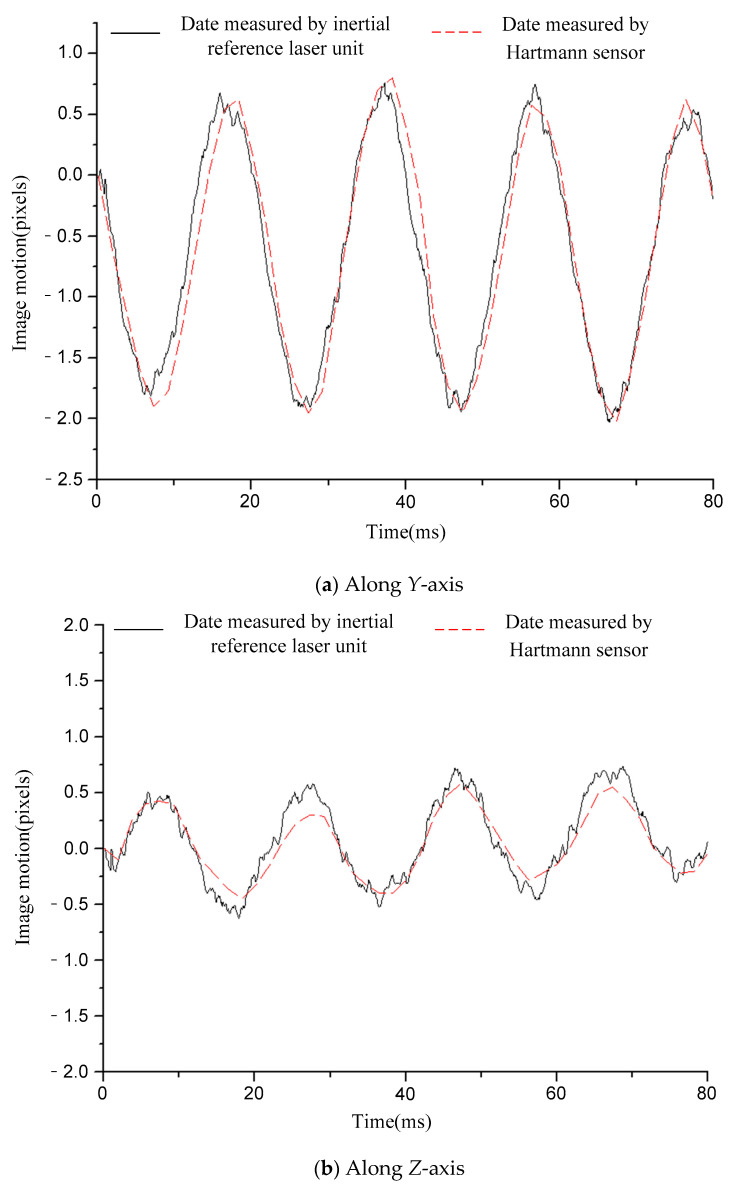
Comparison of image motion measurement curves under the condition of 50 Hz constant frequency sinusoidal excitation.

**Figure 9:**
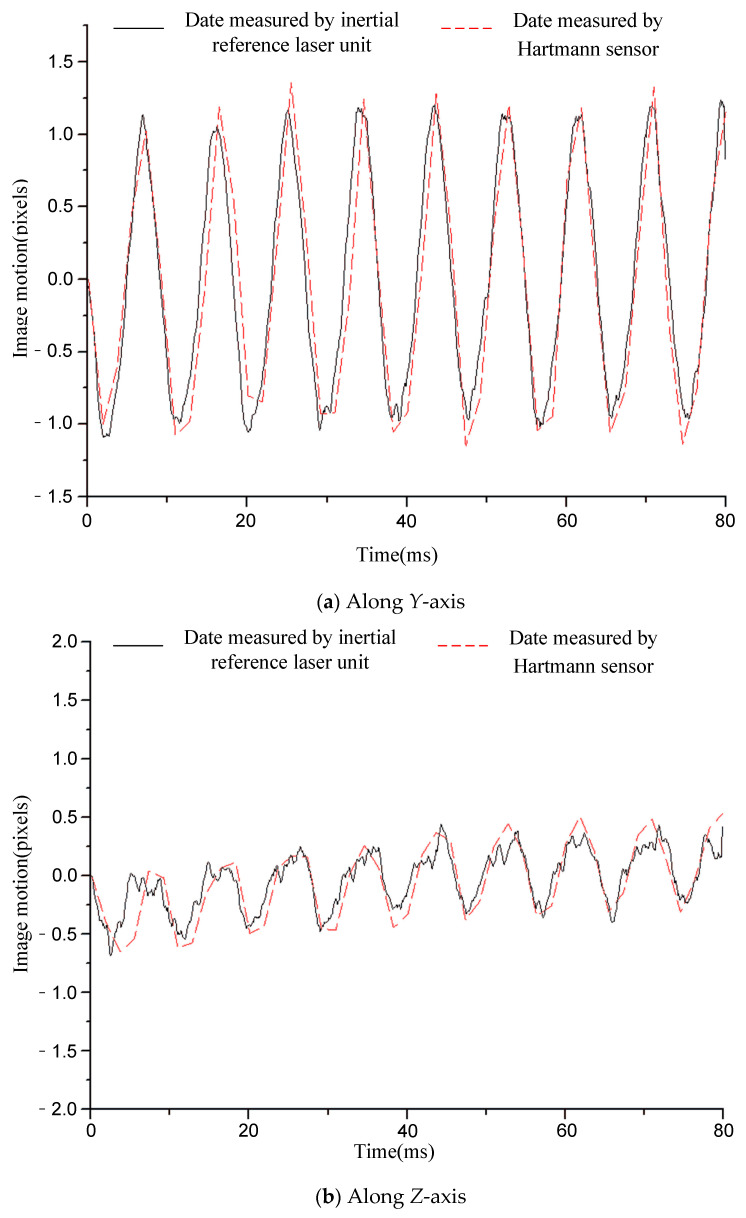
Comparison of image motion measurement curves under the condition of 110 Hz constant frequency sinusoidal excitation.

**Figure 10:**
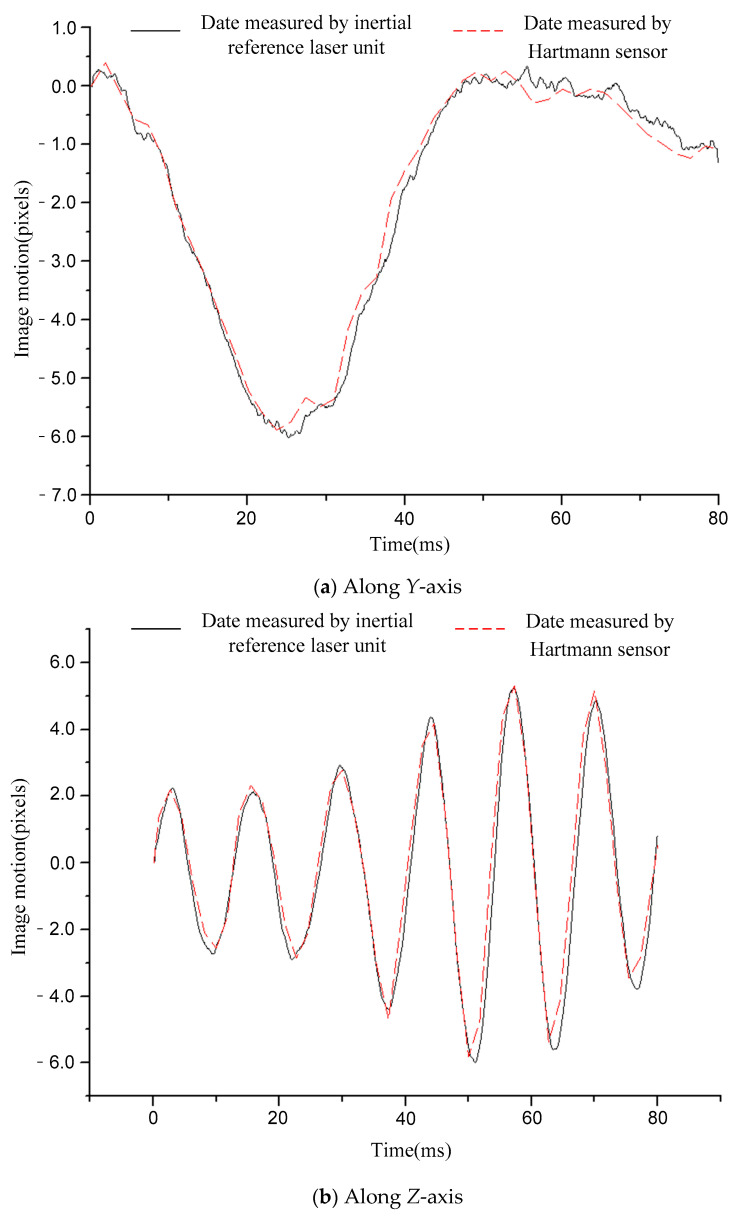
Comparison of image motion measurement curves under the condition of random vibration excitation.

Figure 6, Figure 7, Figure 8, Figure 9 and Figure 10 compare the two curves, from which the following conclusions can be obtained:

(1) The difference between the two curves was less than 0.12 pixels (RMS), see Table 2 for details. The image motion measurement method based on an inertial reference laser reported a high measurement accuracy. In our field, it is acceptable that the image motion measurement accuracy is better than 0.2 pixel. The accuracy of image motion measurement could reach 0.12 pixel, which met the application requirements. The experimental results in Chapter 6 also proved this. (2) The sampling rate of the laser gyroscope and PSD were both 10 kHz, which was higher than that of the Hartmann wavefront sensor (550 Hz). Therefore, the measurement data contained high-frequency information that could precisely describe the image motion process. In addition, because the application of the Hartmann sensor involved image processing, the delay effect was obvious, and it was difficult to feed back the image motion information to the image stabilizer in real time, so it is suitable to store the image motion data for later image restoration. The method based on inertial reference laser proposed in this paper could read the image motion information in real time and feed it back to the image stabilization mirror, which is helpful to obtain a clear image in real time.

For a further explanation of the measurement accuracy, the measurement accuracy of Hartmann sensor was better than 0.1 pixel (RMS), which was the conclusion obtained after a comparison with higher accuracy measurement equipment, but the true value could not be obtained. Compared with the Hartmann sensor, the image motion measurement error of the proposed method was less than 0.12 pixel (RMS).

## 6. Image Quality Improvement Experiment Based on Inertial Reference Laser

An image restoration experiment was performed to further verify the performance and application effect of the image motion measurement method based on an inertial reference laser. The measured image motion information was used to restore a degraded image. After restoration, the MTF value of the degraded and restored images was calculated to quantitatively evaluate the restoration effect.

### 6.1. Experimental Method and Process

**Figure 11:**
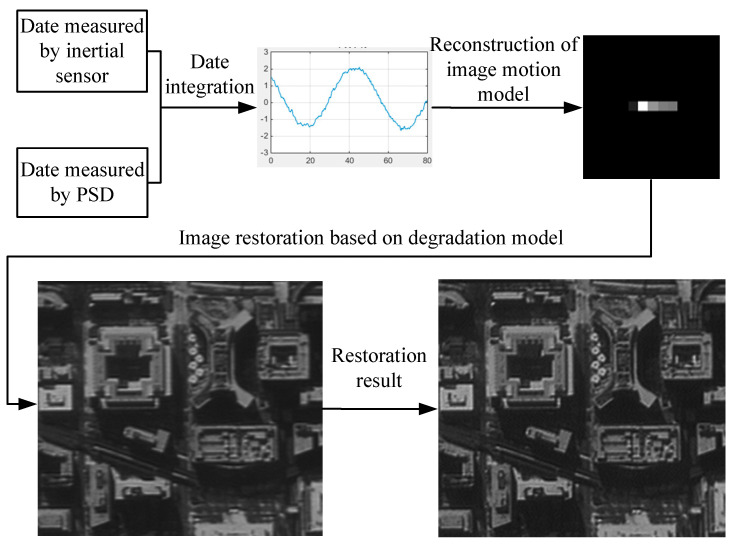
Image restoration method and process based on an inertial reference laser.

The method and process of the image restoration experiment based on an inertial reference laser are illustrated in Figure 11 and are summarized as follows:

(1) A degraded image was taken using the camera, and the inertial reference laser system was used to measure the image motion information of the optical system.
(2) The image motion model was reconstructed based on the image motion information, and the image motion degradation model in the integral time was obtained.
(3) Based on the comprehensive degradation model, the degraded image was restored using image restoration software to obtain a clear image.
(4) The MTF values of the degraded and restored images at a Nyquist frequency were calculated for a quantitative evaluation.

### 6.2. Image Quality Improvement Based on Inertial Reference Laser

**Figure 12:**
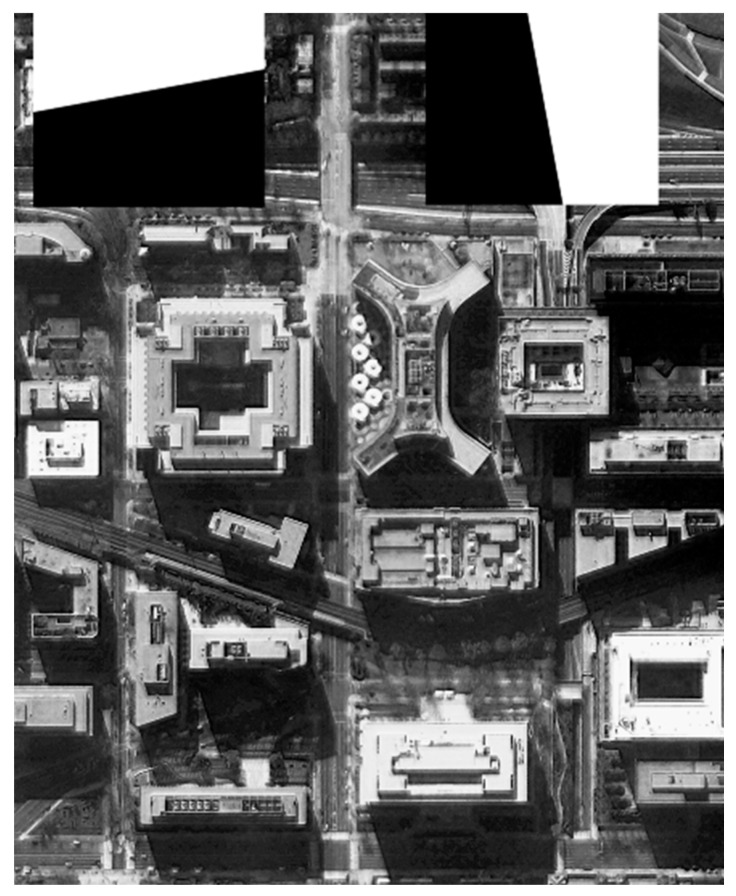
The original image used for the experiment.

The original image shown in Figure 12 was used for the restoration experiment aiming at one architectural image. In order to calculate the MTF of the restored image conveniently, a black-and-white edge was added to the image.

**Figure 13:**
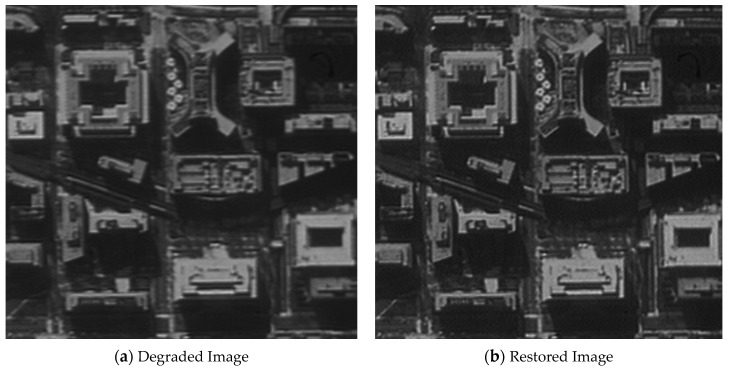
The MTF value is increased from 0.037 to 0.067.

**Figure 14:**
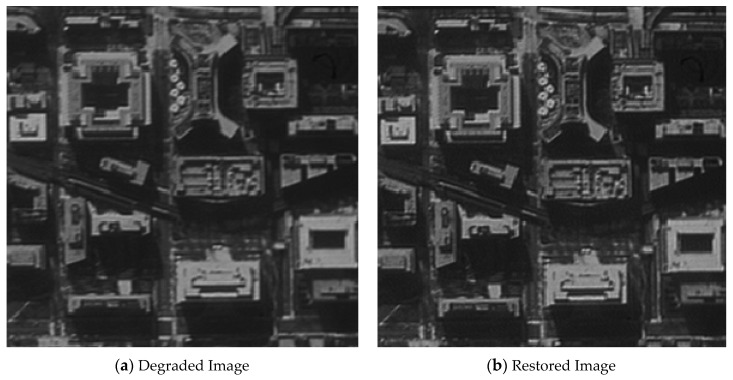
The MTF value is increased from 0.045 to 0.080.

**Figure 15:**
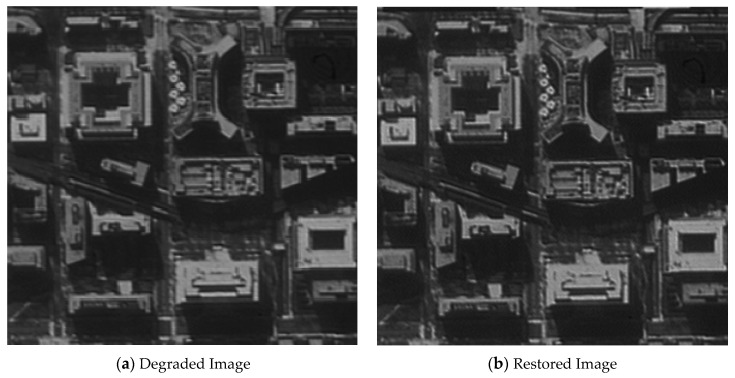
The MTF value is increased from 0.044 to 0.071.

**Figure 16:**
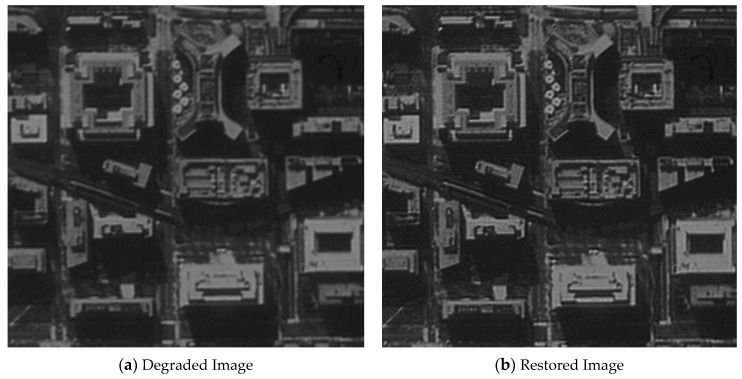
The MTF value is increased from 0.048 to 0.090.

Figure 13, Figure 14, Figure 15 and Figure 16 compare the degraded and restored images under different disturbance conditions. In Figure 13, the experiment condition was set to a 20 Hz constant frequency excitation with a jitter of 3.5 pixels (P-V) and integration time of 80 ms. In Figure 14, the experiment condition was set to a 50 Hz constant frequency excitation, with a jitter of 2.5 pixels (P-V) and integration time of 80 ms.In Figure 15, the experiment condition was set to 110 Hz constant frequency excitation with a jitter of 2.5 pixels (P-V) and integration time of 80 ms. In Figure 16, the experiment condition was random excitation with jitters of 3 pixels (P-V) and 2 pixels (P-V) in the Y and Z directions, respectively, and the integration time was set to 40 ms. The summary table is shown in Table 3.

The visual qualitative evaluation revealed that both the contrast and sharpness of the restored image were significantly improved without obvious ringing, and that the noise was within a controllable range. The MTF of the restored image could be increased to 1.61–1.88 times of the original image. When the image motion was less than 3.5 pixels, the image motion measurement method based on an inertial reference laser could significantly improve the image quality.

## 7. Conclusions

In this paper, an image motion measurement method based on an inertial reference laser was proposed, and an experimental verification system was developed. Based on this system, image motion measurement experiments under various experimental conditions were carried out, and the degraded images were restored using the obtained image motion data. The results show that the image motion measurement method based on an inertial reference laser achieved a higher measurement accuracy with a relative error of less than 0.12 pixels (RMS) compared with the measurement data of the Hartmann wavefront sensor. When the pixel jitter was less than 3.5 pixels, the image motion measurement method based on inertial reference laser significantly improved the image quality and increased the MTF of the image to 1.61–1.88 times of the original image. The obtained image motion data could also be fed back to the image stabilization mirror to compensate for the influence of on-orbit disturbance on the image quality in real time and to effectively improve the image quality of the optical remote sensing satellite. The proposed method has a high measurement accuracy and speed, is not affected by ground lighting conditions, and can be used in other occasions that require a high-precision image motion measurement.

## Figures and Tables

**Table 1:** Root mean square (RMS) value of the background noise of the vibration isolation platform.

	Around *Y*-Axis (″)	Around *Z*-Axis (″)
Background noise (1–200 Hz, RMS)	0.029	0.037
Background noise (3–200 Hz, RMS)	0.014	0.012

**Table 2:** The difference between the two curves (RMS).

Condition		12.5 Hz Sinusoidal	20 Hz Sinusoidal	50 Hz Sinusoidal	110 Hz Sinusoidal	Random Vibration
Difference	*Y*-axis	0.07 pixel	0.07 pixel	0.12 pixel	0.09 pixel	0.08 pixel
	*Z*-axis	0.06 pixel	0.12 pixel	0.11 pixel	0.07 pixel	0.06 pixel

**Table 3:** Summary of the image quality improvement.

Excitation		20 Hz Sinusoidal	50 Hz Sinusoidal	110 Hz Sinusoidal	Random Vibration
Jitter (P-V)		3.5 pixels	2.5 pixels	2.5 pixels	3 pixels (Y) and 2 pixels (Z)
Integration time		80 ms	80 ms	80 ms	40 ms
MTF	Original	0.037	0.045	0.044	0.048
	Increased	0.067	0.080	0.071	0.090

## Data Availability

No new data were created or analyzed in this study. Data sharing is not applicable to this article.
